# Blocking circ-SCMH1 (hsa_circ_0011946) suppresses acquired DDP resistance of oral squamous cell carcinoma (OSCC) cells both in vitro and in vivo by sponging miR-338-3p and regulating LIN28B

**DOI:** 10.1186/s12935-021-02110-8

**Published:** 2021-08-05

**Authors:** Feng Qiu, Bin Qiao, Nan Zhang, Zheng Fang, Lu Feng, Shanfeng Zhang, Weiliu Qiu

**Affiliations:** 1 Department of Stomatology, The First Affiliated Hospital of Zhengzhou University, Zhengzhou, 450052 Henan China; 2 Experimental Center for Basic Medicine, Biochemistry and Molecular Biology, Zhengzhou University, Zhengzhou, 450000 Henan China; 3 School of Basic Medical Sciences, Zhengzhou University, Zhengzhou, 450001 Henan China; 4 Department of Oral and Maxillofacial Surgery, School of Medicine, Ninth People’s Hospital, Shanghai Jiao Tong University, No. 639, Manufacturing Bureau Road, Shanghai, 200011 China

**Keywords:** Circ-SCMH1, miR-338-3p, DDP resistance, OSCC, LIN28B

## Abstract

**Background:**

Circular RNAs (circRNAs) could participate in *cis*-dichlorodiammineplatinum (DDP) resistance of human cancers. However, circRNAs role in DDP resistance of oral squamous cell carcinoma (OSCC) progression remains largely undeveloped. Here, we attempted to explore the role of circ-SCMH1 (ID hsa_circ_0011946) in acquired DDP resistance.

**Methods:**

Expression of circ-SCMH1, microRNA (miR)-338-3p and Lin-28 homolog B (LIN28B) was detected by real-time quantitative PCR and western blotting, and their interactions were confirmed by dual-luciferase reporter assay, RNA immunoprecipitation and RNA pull-down assay. DDP resistance was assessed by MTT assay, colony formation assay, flow cytometry, transwell assays, western blotting, and xenograft experiment. Transmission electron microscopic analysis, nanoparticle tracking analysis and western blotting confirmed the characterizations of extracellular vesicles (EVs).

**Results:**

Circ-SCMH1 was upregulated in DDP-resistant OSCC tissues and cells (SCC-15/DDP and CAL-27/DDP). Circ-SCMH1 knockdown suppressed the half-maximal inhibitory concentration of DDP, colony formation, and migration/invasion in SCC-15/DDP and CAL-27/DDP cells, but promoted apoptosis rate and apoptotic proteins (Bax and cleaved-caspase-3) expression. However, silencing miR-338-3p abrogated above effects, and overexpressing miR-338-3p mimicked that. Similarly, miR-338-3p overexpression role could be counteracted by restoring LIN28B. Moreover, interfering circ-SCMH1 retarded tumor growth of SCC-15/DDP cells in vivo with DDP treatment or not. Mechanistically, circ-SCMH1 directly sponged miR-338-3p in regulating LIN28B, a target gene for miR-338-3p. Notably, circ-SCMH1 was an EVs cargo, and DDP-resistant OSCC cells-derived EVs could provoke circ-SCMH1 upregulation in parental cells.

**Conclusion:**

Circ-SCMH1 contributes to chemoresistance of DDP-resistant OSCC cells partially via EVs secretion and circ-SCMH1/miR-338-3p/LIN28B axis.

**Supplementary Information:**

The online version contains supplementary material available at 10.1186/s12935-021-02110-8.

## Introduction

Oral squamous cell carcinoma (OSCC) is one of the most common (95%) cancer in head and neck squamous cell carcinomas (HNSCCs) that rank the sixth most common malignant cancers [1, 2]. The standard treatment for OSCC is a combination of surgery, radiation, and chemotherapy; cis-diaminodichloroplatinum (DDP) is one first-line chemotherapy drug for HNSCCs [3]. However, many OSCC patients have both innate and acquired DDP resistance [4], thus serving as a major obstacle in the treatment of OSCC [5]. The morbidity and mortality rates of OSCC are high, and the 5-year survival rate is still unsatisfactory [1, 6]. Therefore, it is of great importance to elucidate the molecular mechanism of the malignant development and DDP resistance of OSCC.

Circular RNAs (circRNAs), a portion of RNAs with covalently closed loop, are formed by alternative splicing of pre-messenger RNAs (mRNAs) [7]. The profiles of circRNAs have been screened to be abnormally expressed in tumors of OSCC [8, 9]. Emerging evidences demonstrate that there are correlations between circRNAs and either clinicopathological features of OSCC patients [10, 11] or malignant development of OSCC cells [12]. Furthermore, a few circRNAs have been disclosed to be involved in DDP resistance of different human cancers [13, 14], including OSCC [15]. Very recently, a circRNA derived from SCMH1 (hsa_circ_0011946, circ-SCMH1) has been declared to be highly expressed in human cancers, such as breast cancer [16], hepatocellular carcinoma, and OSCC [17, 18]. However, the precise role of circ-SCMH1 remains largely undisclosed in OSCC.

MicroRNAs (miRNAs) are a subtype of noncoding RNAs and can be isolated from body fluids, such as blood [19]. Circulating miRNAs have been used as diagnostic and prognostic biomarkers for oral cancer [20]. Five miRNAs including miRNA (miR)-338-3p is suggested to possess utility as potential noninvasive biomarkers to detect oral cancer or high-grade lesions [21]. However, the role and regulatory mechanism of miR-338-3p remain to be uncovered in OSCC, especially in chemoresistance.

In this study, we attempted to unravel the role of circ-SCMH1 in acquired DDP resistance in OSCC cells both in vitro and in vivo. Moreover, the competing endogenous RNA (ceRNA) mechanism of circ-SCMH1 was further identified via the regulation of miR-338-3p and Lin-28 homolog B (LIN28B), an RNA-binding protein that closely correlates with OSCC carcinogenesis, aggressiveness and poor prognosis [22].

## Materials and methods

### Patients and tissue samples

A total of 62 primary OSCC patients were recruited in the First Affiliated Hospital of Zhengzhou University, and this study was approved by the Research Scientific Ethics Committee of the First Affiliated Hospital of Zhengzhou University. The tissues samples were obtained during the radical resection surgery after written consent was received from each patient. Prior to operation, all of the cases were treated with DDP and classified into DDP-sensitive or -resistant group according to the clinical treatment record after 2 cycles of DDP (40 mg/m^2^) standard regimens which were given at the 1st and 4th weeks. The evaluation criteria of DDP-sensitive group were tumors with at least 30% decrease in the largest lesion, and the DDP-resistant group was tumors with no significant decrease after DDP treatment. All tissue samples were stored at − 80 °C. Clinicopathologic features of these OSCC patients and their relationships with circ-SCMH1 expression are shown in Table 1.

**Table 1:** Relationship between circ-SCMH1 expression and clinicopathologic features of OSCC patients

	Characteristics (*n* = 62)	circ-SCMH1 expression		*P* value^a^
		Low (*n* = 31)	High (*n* = 31)	
Gender				0.7972
Female	36	17	19	
Male	26	14	12	
Age (years)				0.2831
≤ 50	21	13	8	
> 50	41	18	23	
TNM grade				0.0046*
I + II	28	20	8	
III + IV	34	11	23	
Lymph node metastasis				0.0395*
Positive	35	13	22	
Negative	27	18	9	

*OSCC* oral squamous cell carcinoma, *TNM* tumor node metastasis

*P < 0.05

^a^Chi-square test

### DDP-resistant OSCC cells

The human OSCC cell lines including SCC-15 (code. 0218) and CAL-27 (code. 0326) were purchased from BCRJ cell bank (Beijing, China), and normal human oral keratinocyte (HOK) was from the Cell Bank of Type Culture Collection of Chinese Academy of Sciences (Shanghai, China). SCC-15 and CAL-27 cells were employed to establish DDP-resistant cells through step-by-step suffering DDP (cat. 2251; TOCRIS, Shanghai, China) from 1 μM to 8 μM, and finally suffering 4 μM DDP for a long-term cultivation for 2 month; after that, these cells were defined as SCC-15/DDP and CAL-27/DDP, respectively. All the cells were incubated in DMEM medium (R&D SYSTEMS, Shanghai, China) supplemented with 10% fetal bovine serum (FBS; R&D SYSTEMS) and cultured in a humidified air atmosphere with 5% CO_2_ at 37 °C. Prior to functional assays, SCC-15/DDP and CAL-27/DDP cells were transferred into DDP-free culture medium for 2 days.

### Real-time quantitative PCR (RT-qPCR) and RNase R treatment

Total RNA was isolated from tissues and cells by RNeasy total RNA extraction kit (Qiagen, Valencia, CA, USA). The quantity and quality of isolated RNA were assessed by observing the absorbance at 260 and 280 nm using NanoDrop® (ND1000, Thermo Scientific, Delaware City, Delaware, USA), and A260/A280 ratio was 1.9–2.1. RNA integrity number (RIN) values were determined on an Agilent 2100 Bioanalyzer using Expert software (Rev. B.02.08.SI648) [23]. RIN values of total RNA were 7.6–9.2. An aliquot (2 μg) of total RNA was followed with 3 U/μg of RNase R treatment for 20 min at 37 °C. For miRNA analysis, RNAs were extracted using a miRNeasy Mini kit (Qiagen), and A260/A280 ratio was 1.8–2.2 and RIN value was 7.1–8.6. The cDNA was synthesized by Prime Script RT Master Mix reagent kit with gDNA Eraser (TaKaRa, Kyoto, Japan), and further amplified using SYBR Green Master Mix (TaKaRa). The PCR amplification was performed and analyzed on 7500 Real-Time PCR system (Applied Biosystems, Foster City, California, USA). Primer sequence was listed as follows: hsa_circ_0000199 (circ_0000199) forward CATTGCTTTCAGGGCTCTTGA and reverse CCGCTCTCTCGACAAATGGA, circ_0109291 forward CCCGATCACCAGTCTAGAGC and reverse GCATTCCCACTCCTCCAGA, circ_0001821 forward TGTAAGACCCCGACTCTTCC and reverse CCATCTTGAGGGGCATCTTT, circ_0013339 forward GCTCTAGATCAGGCACATGG and reverse CCAAAATGGCAGTGCTATCGA, circ-SCMH1 forward CTCCCGAGACATCTTCCCTG and reverse CACTAGATGCTTTGGTGCCA, SCMH1 forward AGAAGCTGCTGACATCCTGG and reverse AGCCGCTGAACAGAATGAAA, miR-338-3p forward TCCAGCATCAGTGATT and reverse GTGCAGGGTCCGAGGT, LIN28B forward GAAGGCGGGGCTAGCAA and reverse CATGCGCACATTGAACCACT, glyceraldehyde-phosphate dehydrogenase (GAPDH) forward AGAAGGCTGGGGCTCATTTG and reverse AGGGGCCATCCACAGTCTTC, β-actin forward TCACCATGGATGATGATATCGC and reverse ATAGGAATCCTTCTGACCCATGC, and U6 forward GGAACGATACAGAGAAGATTAGC and reverse TGGAACGCTTCACGAATTTGCG. Gene expression was analyzed using the 2^−ΔΔCt^ method (Ct, cycle threshold), and the experimental gene expression was normalized to internal control (U6 for miRNA or GAPDH/β-actin for circRNA and mRNA). The primers for circ-SCMH1 were designed in circPrimer 1.2.0.5 software [24]. For subcellular location assay, the nuclear and cytoplasmic fractions were isolated by the PARIS kit (Invitrogen, Carlsbad, CA, USA) following the manufactures’ protocols.

### Cell transfection

To construct overexpression vector, the full lengths of circ-SCMH1 and LIN28B cDNA were separately cloned into pcDNA3.1/Hygro (+) vector (pcDNA; Invitrogen). The oligonucleotides including siRNA (shRNA) against circ-SCMH1 (si (sh)-circ-SCMH1), miR-338-3p mimic, and anti-RNA against miR-338-3p (anti-miR-338-3p) were synthesized by GENEWIZ (Beijing, China), as well as the negative controls. For cell transfection, vectors (2 μg) and oligonucleotides (40 nM) were transfected into SCC-15/DDP and CAL-27/DDP cells (in 6-well plate) using Lipofectamine RNA iMAX (Invitrogen) according to the instruction. The sequence of these oligonucleotides was si-circ-SCMH1 ACCAAAGCATCTAGTGCTTTT, miR-338-3p mimic UCCAGCAUCAGUGAUUUUGUUG, anti-miR-338-3p CAACAAAATCACTGATGCTGGA, si-NC CCTAAGGTTAAGTCGCCCTCG, miR-NC mimic UUUGUACUACACAAAAGUACUG, and anti-miR-NC UCUACUCUUUCUAGGAGGUUGGA. The pSilencer2.1-U6 hygro (EK-Bioscience, Shanghai, China) was used for expressing shRNAs and stable transfection in SCC-15/DDP cells. The sequence of sh-circ-SCMH1 with hairpin sequence was AGCTGCACCAAAGCATCTAGTGCTTTCAAGAGAAGCACTAGATGCTTTGGTGCTTTTTG and GATCCAAAAAGCACCAAAGCATCTAGTGCTTCTCTTGAAAGCACTAGATGCTTTGGTGC, and sh-NC with hairpin sequence was AGCTCCTAAGGTTAAGTCGCCCTCGTCAAGAG CGAGGGCGACTTAACCTTAGGTTTTTG and GATCCAAAAACCTAAGGTTAAGTCGCCCTCGCTCTTGACGAGGGCGACTTAACCTTAGG. After transfection for 48 h, SCC-15/DDP and CAL-27/DDP cells were harvested for further analysis.

### MTT assay

SCC-15/DDP and CAL-27/DDP cells after transfection or not, as well as the parental cells, were exposed to different concentrations of DDP ranging from 0 to 40 μM for 48 h prior to cell viability analysis. MTT reagent (2 mg/mL) was added to each well after discarding medium. With 4 h incubation, 100 μL of dimethylsulfoxide was added after discarding medium containing MTT. The optical density (OD) was read at 450 nm/570 nm using a microplate reader after shaking for 10 min to represent cell viability, and dose response curve was drawn. The half-maximal inhibitory concentration (IC_50_) of DDP was calculated with GraphPad Prism Version 5.0 Software (GraphPad, San Diego, CA, USA).

### Colony formation assay

SCC-15/DDP and CAL-27/DDP cells after transfection or not were transferred to new 6-well plate to a concentration of 300 cells/well. The cell suspension was uniformly distributed with single cells, and the fresh culture medium was replaced every 3 days. After 15 days, the cell colonies were washed twice with phosphate-buffered saline (PBS) for twice, fixed with 4% paraformaldehyde for 30 min and stained with 0.1% crystal violet for 30 min. The colonies were photographed, and the number of colonies was counted by ImageJ software (National Institutes of Health, Bethesda, MD, USA). The number of colonies (more than 50 cells) was counted under a microscope.

### Flow cytometry (FCM)

SCC-15/DDP and CAL-27/DDP cells after transfection or not were collected and re-suspended. The cells were then stained with AnnexinV-FITC Apoptosis Detection Kit I (BD Biosciences, San Jose, CA, USA) following the manufacturer’s instruction. The apoptotic cells were discriminated on FACS Vantage SE flow cytometer (BD Biosciences) with the dual excitation wavelength of 488 nm and 510 nm, and apoptosis rate (%) was calculated on FlowJo software (BD Biosciences). Stained cells were analyzed on Annexin V-FITC/PI quadrants, and apoptosis rate was calculated as the percentage of Annexin V-FITC + /PI- and Annexin V-FITC + /PI + cells.

### Transwell assays

SCC-15/DDP and CAL-27/DDP cells after transfection or not were collected to measure abilities of migration and invasion using Transwell chambers (Corning, NY, USA). For migration assay, serum-starved cells (2 × 10^4^) were incubated in DMEM (without FBS) and then seeded in the upper chamber (in 24-well plate), 500 μL of culture medium containing 10% FBS was added to the lower chamber. This transwell system was cultivated in normal cell culture condition for another 24 h. Next, the migrated cells on the bottom of chamber were fixed with 4% paraformaldehyde for 30 min and stained with 0.1% crystal violet for 30 min. The images of cells were captured under a microscope (100×), and migrated cell number was calculated according to 5 randomly selected fields. For invasion assay, transwell chambers were pre-coated with 50 µl of growth factor-reduced Matrigel (BD Biosciences), and 2 × 10^5^ cells were used; the other performances were the same to migration assay.

### Dual-luciferase reporter assay

miRNA binding sites in circ-SCMH1 and LIN28B were predicted by circinteractome algorithm [25] (https://circinteractome.nia.nih.gov/api/v2/mirnasearch?circular_rna_query=hsa_circ_0011946&mirna_query=&submit=miRNA+Target+Search) and starBase v2.0 algorithm [26] (http://starbase.sysu.edu.cn/agoClipRNA.php?source=mRNA&flag=target&clade=mammal&genome=human&assembly=hg19&miRNA=all&clipNum=1&deNum=0&panNum=0&proNum=1&program=&target=LIN28B), respectively. Briefly, enter the ID of circ-SCMH1 (hsa_circ_0011946) in Step 1, enter the ID of miR-338-3p (hsa-miR-338-3p), and click on “miRNA Target Search” button in circinteractome; in starBase, click the miRNA–mRNA interaction in the pull-down list box, enter the ID of miR-338-3p (hsa-miR-338-3p), and click the Submit button, and consequently LIN28B was one of the results. The intact primary sequence of circ-SCMH1 (782 bp) containing the putative binding sites (AUGCUGG) were sub-cloned from overexpression vectors into psiCHECK-2 luciferase report vectors to generate WT-circ-SCMH1 vectors. And, putative binding sites in WT-circ-SCMH1 vector were site-mutated into UACGACC using QuickMutation™ Site-Directed Mutagenesis Kit (Beyotime, Shanghai, China) to generate MUT-circ-SCMH1 vectors. Similarly, the 3′untranslated region (3′UTR) of LIN28B was based on to establish WT-LIN28B 3′UTR and MUT-LIN28B 3′UTR. SCC-15/DDP and CAL-27/DDP cells were co-transfected with WT/MUT vector (400 ng) and miR-338-3p mimic or miR-NC mimic (40 pM) for 48 h. The luciferase activities in cell lysates were detected on GloMax®-Multi + Luminescence Module (Promega, Madison, WI, USA).

### RNA immunoprecipitation (RIP) and RNA pull-down assay

EZ-magna RIP kit (Millipore, Billerica, MA, USA) was used to perform argonaute 2 (Ago2) RIP with the normalization to negative control immunoglobulin G (IgG) RIP. In brief, cell extracts of SCC-15/DDP and CAL-27/DDP cells were obtained and incubated with magnetic beads pre-coated with antibody against to Ago2 (Anti-Ago2; ab32381, 1:50, Abcam) or the control mouse Anti-IgG (ab2410, 1:50, Abcam). Then, precipitated complex was lysed in Trizol (Invitrogen) to detect RNA levels of circ-SCMH1, miR-338-3p and LIN28B using RT-qPCR. The chemosynthetic biotinylated miR-338-3p (bio-miR-338-3p) and biotin-labeled miR-NC (bio-miR-NC) were synthesized by GENEWIZ, and then transfected into SCC-15/DDP and CAL-27/DDP cells for 2 days. Cell extract of bio-miRNAs-transfected cells was collected in RIP lysis buffer, and was next incubated with Streptavidin MagneSphere Paramagnetic beads (Promega). The pull-down complex was lysed in Trizol (Invitrogen) to detect RNA levels of circ-SCMH1 and LIN28B using RT-qPCR.

### Western blotting

Tissues and cells were lysed in radioimmunoprecipitation reagent (RIPA; Invitrogen) with centrifugation at 12,000 g for 15 min, and the supernatant was collected as total protein. A portion of total protein (20 μg) was separated by SDS-polyacrylamide gel electrophoresis and blotted onto polyvinylidene fluoride (Millipore). The primary antibodies target LIN28B (ab229628; 1:2 500), CD63 (ab118307; 1:1 000), CD9 (ab223052; 1:1 000), Calnexin (ab75801; 1:2,000), Bcl2-associated X protein (Bax; ab270742; 1:1,000), cleaved-caspase-3 (ab2302; 1:500), and GAPDH (ab9485; 1:2 500) were provided by abcam (Shanghai, China), as well as the secondary antibody Goat Anti-Rabbit IgG H&L (HRP) (ab205718; 1:50 000). Band intensity was assessed using an ECL reagent (Millipore) and analyzed on ImageJ software (National Institutes of Health).

### Xenograft tumor models

Xenograft experiment was performed in BALB/c nude mice (4–6 weeks old, male) divided into four groups (*n* = 6): sh-NC + PBS, sh-NC + DDP, sh-circ-SCMH1 + PBS, and sh-circ-SCMH1 + DDP. These mice were purchased from the Laboratory Animal Center of Shanghai Academy of Science (Shanghai, China). This animal experiment was approved by National Institute of Health Guide for the Care and Use of Laboratory Animals under the approval of the SPF Laboratory Animal Center at the First Affiliated Hospital of Zhengzhou University. SCC-15/DDP cells were stably transfected with sh-circ-SCMH1 or sh-NC vectors, and then a portion of cell suspension (5.0 × 10^6^ cells) was subcutaneously injected into the back of nude mice. After implantation for 8 days, mice were treated with DDP (3 mg/kg) or equal volume of PBS via intraperitoneal injection every 3 days. With 7 times of DDP treatment, xenograft mice were euthanatized and tumors were resected. During animal feeding, the sizes of tumors were measured with caliper at indicated times, and the volume was calculated using 0.5 × [length] × [width]^2^ equation. The tumor weight was recorded using electronic balance on the last day. All the operations were performed following the Guide for the Care and Use of Laboratory Animals of the National Institutes of Health.

### Extracellular vesicles (EVs) isolation and identification

SCC-15/DDP and CAL-27/DDP cells were cultured in exosome-depleted FBS (SBI, Palo Alto, CA, USA) for 2 days and then the supernatant was harvested for EVs isolation using ExoQuick-TC Kit (SBI) according to manufacturer’s instructions. The extracted EVs were dissolved in PBS and further verified by transmission electron microscope (TEM) (Hitachi, Tokyo, Japan), NanoSight LM10 instrument (Malvern Panalytical, Malvern, UK) and western blotting. The image analysis of nanoparticle tracking analysis (NTA) software suite was used to automatically track particle size, and five video times of 60 s were taken for each sample. Data were analyzed using the NTA 3.0 software, and hydrodynamic diameters of each particle were calculated using the Stokes–Einstein equation: *D* = *kT/6πηr*, where *D* is the diffusion coefficient, *k* is Boltzmann’s constant, and *T* is the absolute temperature, *r* is the radius of the particle, and *η* is the viscosity of the fluid, which means a spherical particle moving with the uniform velocity in a continuous fluid. Another portion of EVs were lysed in Trizol (Invitrogen) for extracellular RNA isolation and lysed in RIPA (Beyotime) for EVs protein isolation. Some EVs were incubated with 1 μg/mL RNase A (Sigma-Aldrich, Cambridge, MA, USA) alone or together with 0.1% Triton X100 (Beyotime) for 30 min prior to EVs RNA isolation.

### EVs treatment

The isolated EVs (100 μg/mL) were added in the fresh medium of SCC-15 and CAL-27 cells for 48 h, and equal volume of PBS was added as control. Total RNA was isolated after EVs/PBS treatment, and cellular circ-SCMH1 expression was detected using RT-qPCR.

### Statistical analysis

The data were collected from three separate assays, and presented as mean ± standard deviation. The data between two groups were analyzed using unpaired Student’s *t* test, and the data among multiple groups were analyzed using one-way analysis of variance, followed by Tukey’s post hoc test. Pearson correlation (*r*) analysis was also performed. *P*-values less than 0.05 were determined to be significant. All the data analysis was performed on GraphPad Prism Version 5.0 Software (GraphPad, La Jolla, CA, USA).

## Results

### Circ-SCMH1 was upregulated in DDP-resistant OSCC tissues and cells

**Fig. 1:**
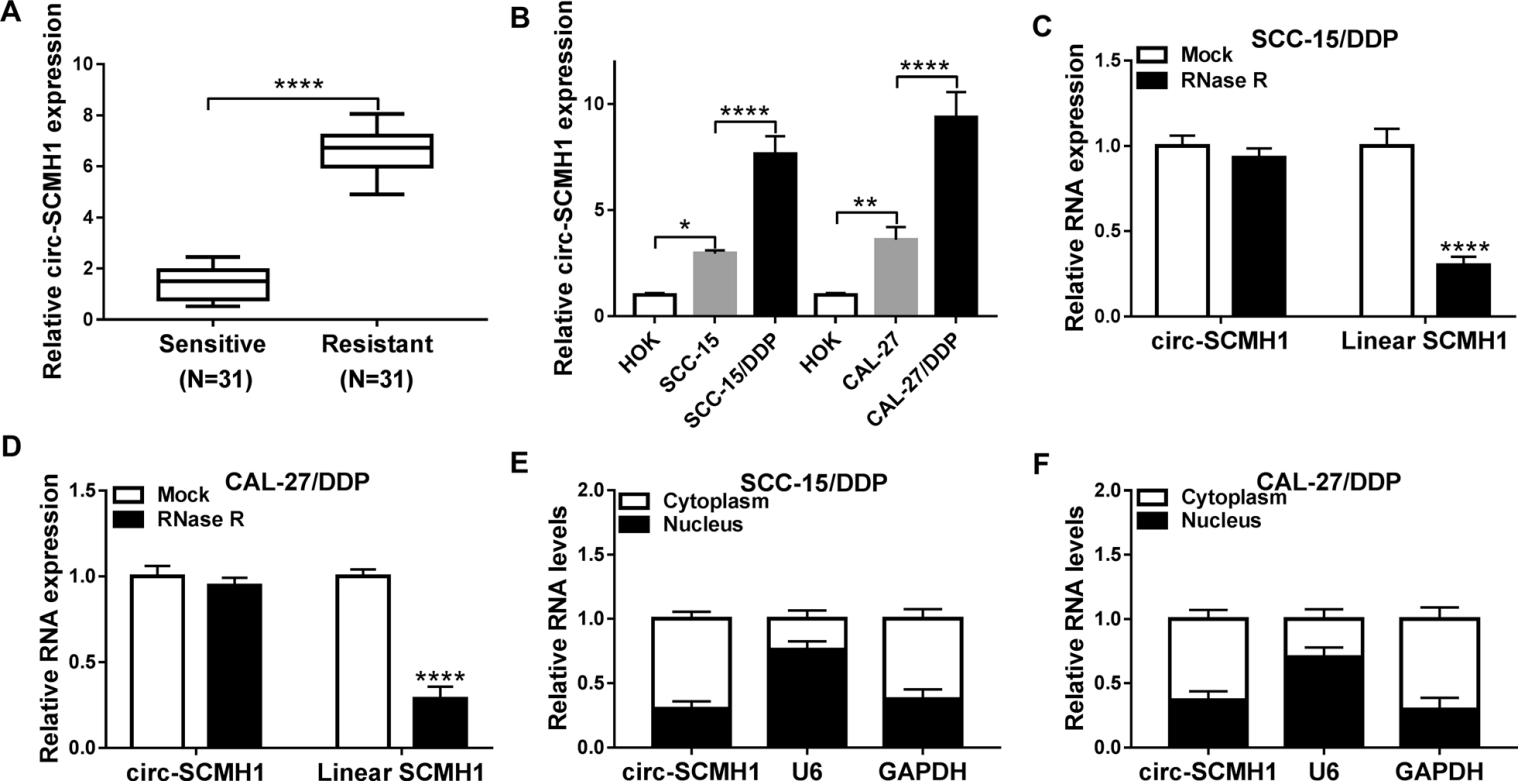
Expression of circRNA derived from SCMH1 (hsa_circ_0011946, circ-SCMH1) in *cis*-dichlorodiammineplatinum (DDP)-resistant oral squamous cell carcinoma (OSCC) tissues and cells. RT-qPCR detected circ-SCMH1 level with normalization to GAPDH in **A** DDP-resistant tumor tissues (Resistant; *N* = 31) and DDP-sensitive tumor tissues (Sensitive; *N* = 31), and **B** DDP-resistant OSCC cell lines (SCC-15/DDP and CAL-27/DDP) and the parental cell lines (SCC-15 and CAL-27), compared to normal human oral keratinocytes (HOK). In SCC-15/DDP and CAL-27/DDP cells, **C–F** RT-qPCR examined RNA levels of **C**, **D** circ-SCMH1 and linear SCMH1 after RNase R treatment or Mock treatment and **E**, **F** circ-SCMH1, U6 and GAPDH in the cytoplasm and nucleus with normalization to GAPDH. *P < 0.05, **P < 0.01, and ****P < 0.0001 from three separate assays

According to RT-qPCR, expression of circ_0109291, circ_0001821 and circ_0011946 (namely circ-SCMH1) was validated to be abnormally upregulated in these 31 DDP-resistant OSCC tissues (Additional file 1: Figure S1), and circ-SCMH1 was the most highly upregulated one. Thus, circ-SCMH1 was selected for further study. Circ-SCMH1 expression was associated with TNM grade and lymph node metastasis of OSCC patients (Table 1). Moreover, level of circ-SCMH1 was 6.6-fold higher in DDP-resistant OSCC tumor tissues than that in DDP-sensitive ones (Fig. 1A and Additional file 2: Figure S2A). Besides, circ-SCMH1 was upregulated in OSCC cell lines (SCC-15 and CAL-27) versus normal HOK cell line, and was even higher in DDP resistance-acquired OSCC cell lines (SCC-15/DDP and CAL-27/DDP) than parental cells (Fig. 1B and Additional file 2: Figure S2B). Comparing to linear SCMH1, circ-SCMH1 expression was little decreased by RNase R treatment in both SCC-15/DDP and CAL-27/DDP cells (Fig. 1C and D and Additional file 2: Figure S2C-S2D). Moreover, circ-SCMH1 expression was predominantly discovered in the cytoplasm, which was paralleled with GAPDH and opposite to U6 (Fig. 1E and F and Additional file 2: Figure S2E and S2F). These results indicated that circ-SCMH1 was stably upregulated in OSCC tumor tissues and cells.

### Knockdown of circ-SCMH1 suppressed chemoresistance and cell progression of DDP-resistant OSCC cells in vitro

**Fig. 2:**
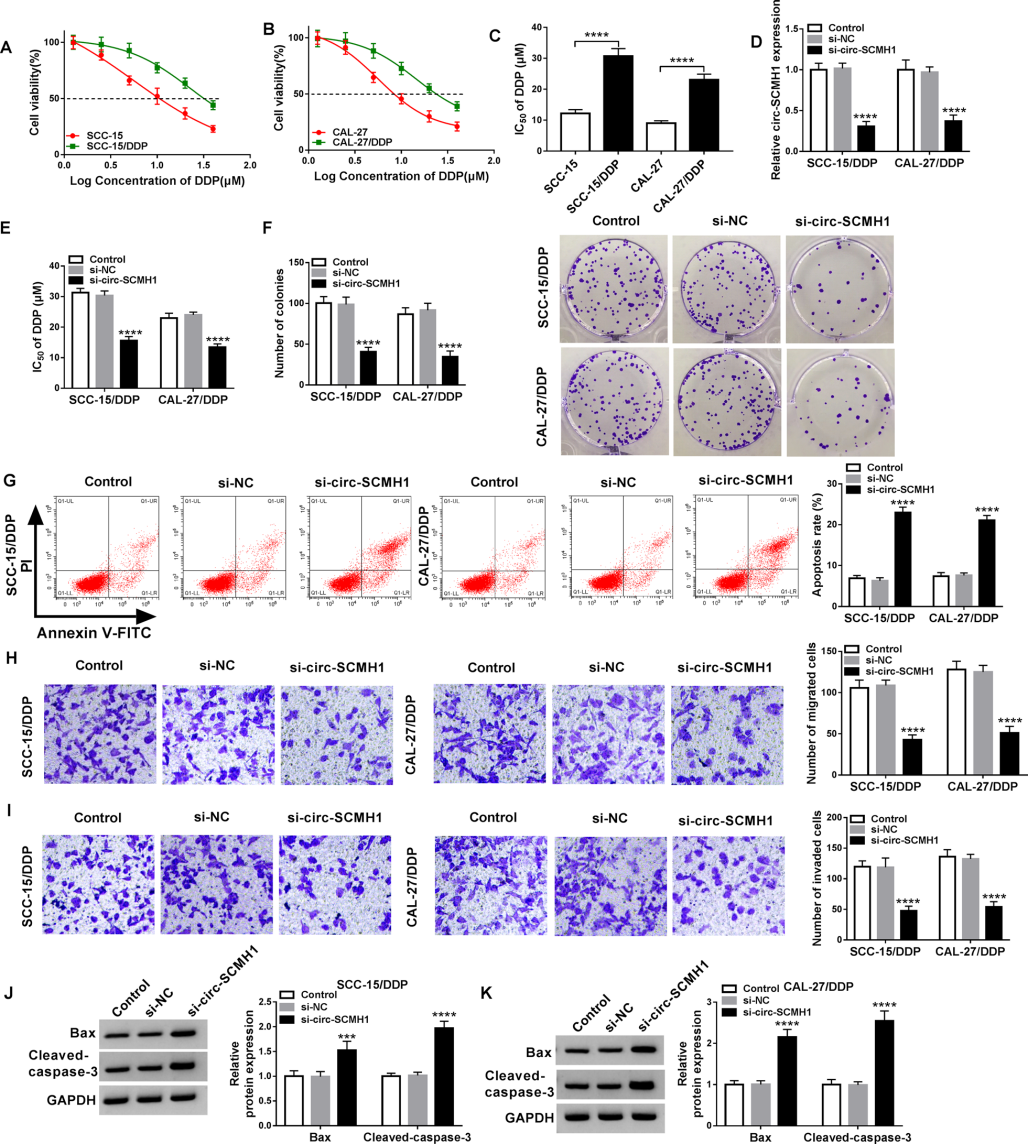
The role of circ-SCMH1 in DDP resistance of OSCC cells in vitro. **A**, **B** MTT assay measured cell viability of SCC-15, SCC-15/DDP, CAL-27, and CAL-27/DDP cells. **C** The half-maximal inhibitory concentration (IC_50_) of DDP was determined in the dose response curves. **D**–**I** SCC-15/DDP and CAL-27/DDP cells were transfected with siRNA against circ-SCMH1 (si-circ-SCMH1) or negative control (si-NC) for 48 h, comparing to control cells (without transfection). **D** RT-qPCR detected circ-SCMH1 level with normalization to GAPDH. **E** MTT assay determined IC_50_ of DDP. **F** Colony formation assay measured number of colonies. **G** Flow cytometry (FCM) analyzed apoptosis rate. **H**, **I** Transwell assays measured numbers of migrated cells and invaded cells. **J**, **K** Western blotting examined relative protein expression of Bax and cleaved-caspase-3 with correction with GAPDH. ***P < 0.001 and ****P < 0.0001 from three separate assays

SCC-15/DDP and CAL-27/DDP cells were established, and MTT assay demonstrated that DDP treatment suppresses cell viability of both parental cells and SCC-15/DDP and CAL-27/DDP cells (Fig. 2A and B); however, SCC-15/DDP and CAL-27/DDP cells showed an increase of IC_50_ (about 2.5-fold) of DDP (Fig. 2C). This suggested an acquired DDP resistance in SCC-15/DDP and CAL-27/DDP cells. Expression of circ-SCMH1 was artificially silenced using siRNA transfection, and si-circ-SCMH1 resulted in a dramatic decrease of circ-SCMH1 in SCC-15/DDP and CAL-27/DDP cells without alteration of its host gene expression (Fig. 2D and Additional file 3: Figure S3). Circ-SCMH1 expression was maintained to be silenced after its siRNA transfection till 4 days (Additional file 4: Figure S4A). With circ-SCMH1 knockdown, IC_50_ values of DDP in SCC-15/DDP and CAL-27/DDP cells were significantly lowered (Fig. 2E). Meanwhile, colony formation numbers of SCC-15/DDP and CAL-27/DDP cells were reduced by silencing circ-SCMH1 (Fig. 2F), accompanied with elevated apoptosis rate (Fig. 2G) and promoted expression of Bax and cleaved-caspase-3 (Fig. 2J and K). Transwell assay showed that numbers of migrated and invaded cells were declined due to circ-SCMH1 deficiency (Fig. 2H and I). Above-mentioned data depicted a suppressive role of circ-SCMH1 knockdown in acquired chemoresistance in DDP-resistant OSCC cells in vitro. Accidently, silencing of circ-SCMH1 suppressed cell progression of the parental cells (DDP-sensitive OSCC cells), as well (Additional file 5: Figure S5A–S5F).

### Circ-SCMH1 functioned as a sponge of miR-338-3p in OSCC cells

**Fig. 3:**
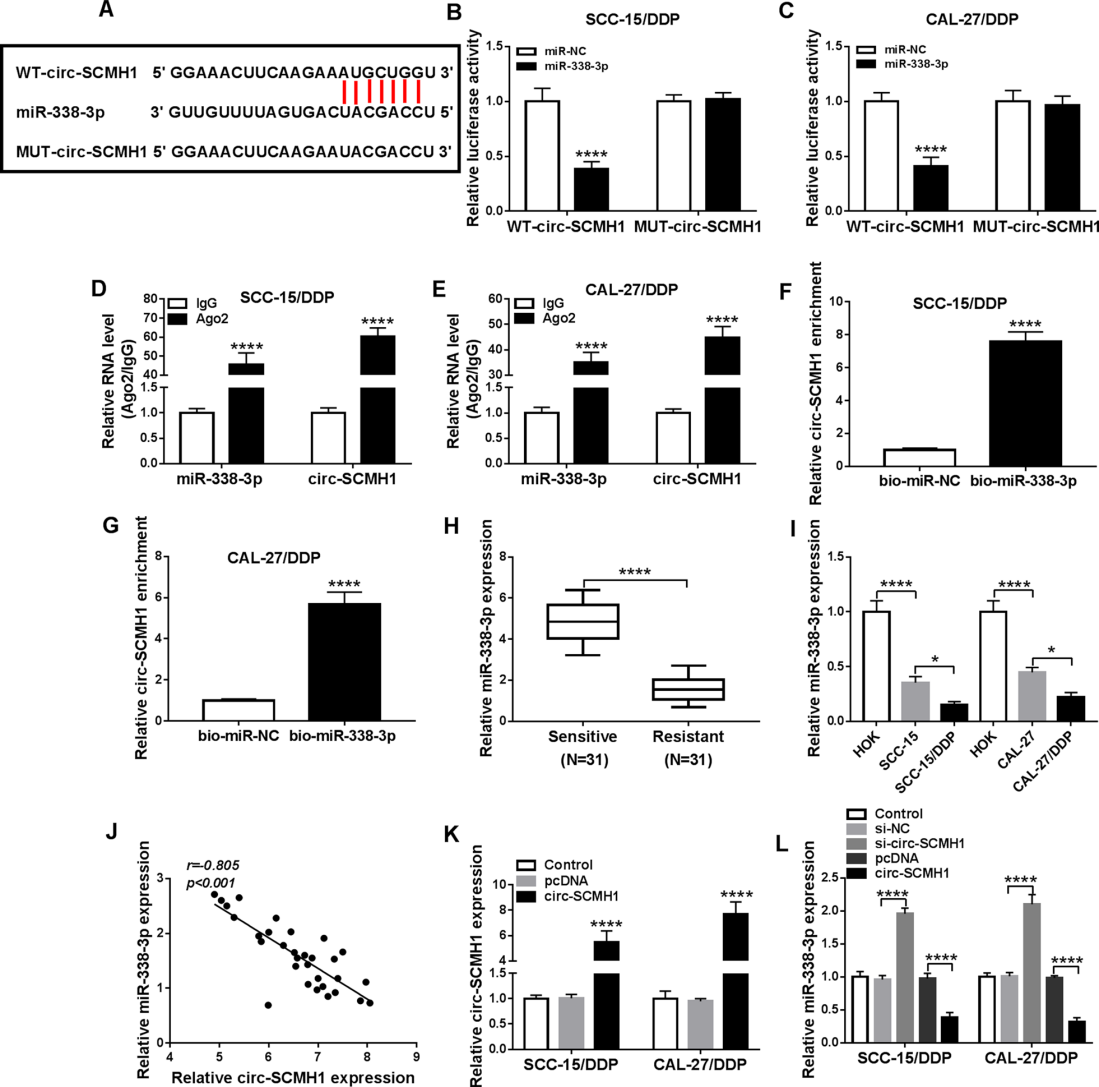
Circ-SCMH1 functioned as a sponge of miR-338-3p in OSCC cells. **A** The circinteractome algorithm showed the complementary binding sites among wild-type of circ-SCMH1 (WT-circ-SCMH1), miR-338-3p and mutant-type of circ-SCMH1 (MUT-circ-SCMH1). **B**, **C** Dual-luciferase reporter assay examined luciferase activity of SCC-15/DDP and CAL-27/DDP cells co-transfected with WT/MUT-circ-SCMH1 and miR-338-3p mimic (miR-338-3p) or the negative control (miR-NC). **D**, **E** RNA immunoprecipitation (RIP) assays of IgG and Ago2 examined RNA levels of circ-SCMH1 and miR-338-3p in cell extracts of SCC-15/DDP and CAL-27/DDP cells. **F**, **G** RNA pull-down assay measured circ-SCMH1 enrichment in cell extracts of SCC-15/DDP and CAL-27/DDP cells transfected with biotin-labelled miR-338-3p (bio-miR-338-3p) or bio-miR-NC. **H**, **I** RT-qPCR measured miR-338-3p level in OSCC tissues in Resistant and Sensitive groups (*N* = 31) and cell lines HOK, SCC-15, SCC-15/DDP, CAL-27, and CAL-27/DDP with normalization to GAPDH. **J** Pearson’s correlation (*r*) analysis evaluated the relationship between circ-SCMH1 and miR-338-3p expression in DDP-resistant OSCC tissues (*N* = 31). **K** RT-qPCR detected circ-SCMH1 level in SCC-15/DDP and CAL-27/DDP cells transfected with circ-SCMH1-overexpression vector (circ-SCMH1) or vehicle vector (pcDNA) with normalization to GAPDH. **L** RT-qPCR detected miR-338-3p level in SCC-15/DDP and CAL-27/DDP cells transfected with si-NC, si-circ-SCMH1, pcDNA, or circ-SCMH1 with normalization to U6. *P < 0.05 and ****P < 0.0001 from three separate assays

Subsequently, the molecular mechanism of circ-SCMH1 in OSCC cells was further identified through serving as miRNAs sponge. The circinteractome algorithm (https://circinteractome/hsa_circ_0011946-mirna) provided a potential binding relationship between circ-SCMH1 and miR-338-3p (Fig. 3A). Dual-luciferase reporter assay confirmed an attenuated luciferase activity of SCC-15/DDP and CAL-27/DDP cells co-transfected with WT-circ-SCMH1 vector and miR-338-3p mimic (Fig. 3B and C), and a stable luciferase activity with co-transfection of MUT-circ-SCMH1 vector and miR-338-3p mimic (Fig. 3B and C). MiR-338-3p mimic could overexpress miR-338-3p till 4 days after transfection (Additional file 4: Figure S4B). Furthermore, RIP assay demonstrated that circ-SCMH1 and miR-338-3p were concurrently enriched in Ago2-mediated precipitation (Fig. 3D and E), and RNA pull-down assay revealed an enrichment of circ-SCMH1 in bio-miR-338-3p-mediated precipitation (Fig. 3F and G). These outcomes suggested miR-338-3p as a target of circ-SCMH1. Expression of miR-338-3p was low expressed in DDP-resistant OSCC tumor tissues and cells (SCC-15/DDP and CAL-27/DDP) in contrast with DDP-sensitive ones (Fig. 3H and I). Moreover, miR-338-3p expression in DDP-resistant OSCC patients was negatively correlated to circ-SCMH1 (Fig. 3J). RT-qPCR analysis also revealed that circ-SCMH1 overexpression via pcDNA vector transfection led to lower level of miR-338-3p in SCC-15/DDP and CAL-27/DDP cells (Fig. 3K and L); contrarily, circ-SCMH1 deficiency via siRNA transfection caused higher level of miR-338-3p (Fig. 3L). These outcomes indicated a reciprocal role of circ-SCMH1 and miR-338-3p in OSCC cells via sponging.

### Circ-SCMH1 deficiency suppressed chemoresistance of DDP-resistant OSCC cells in vitro by liberating miR-338-3p

**Fig. 4:**
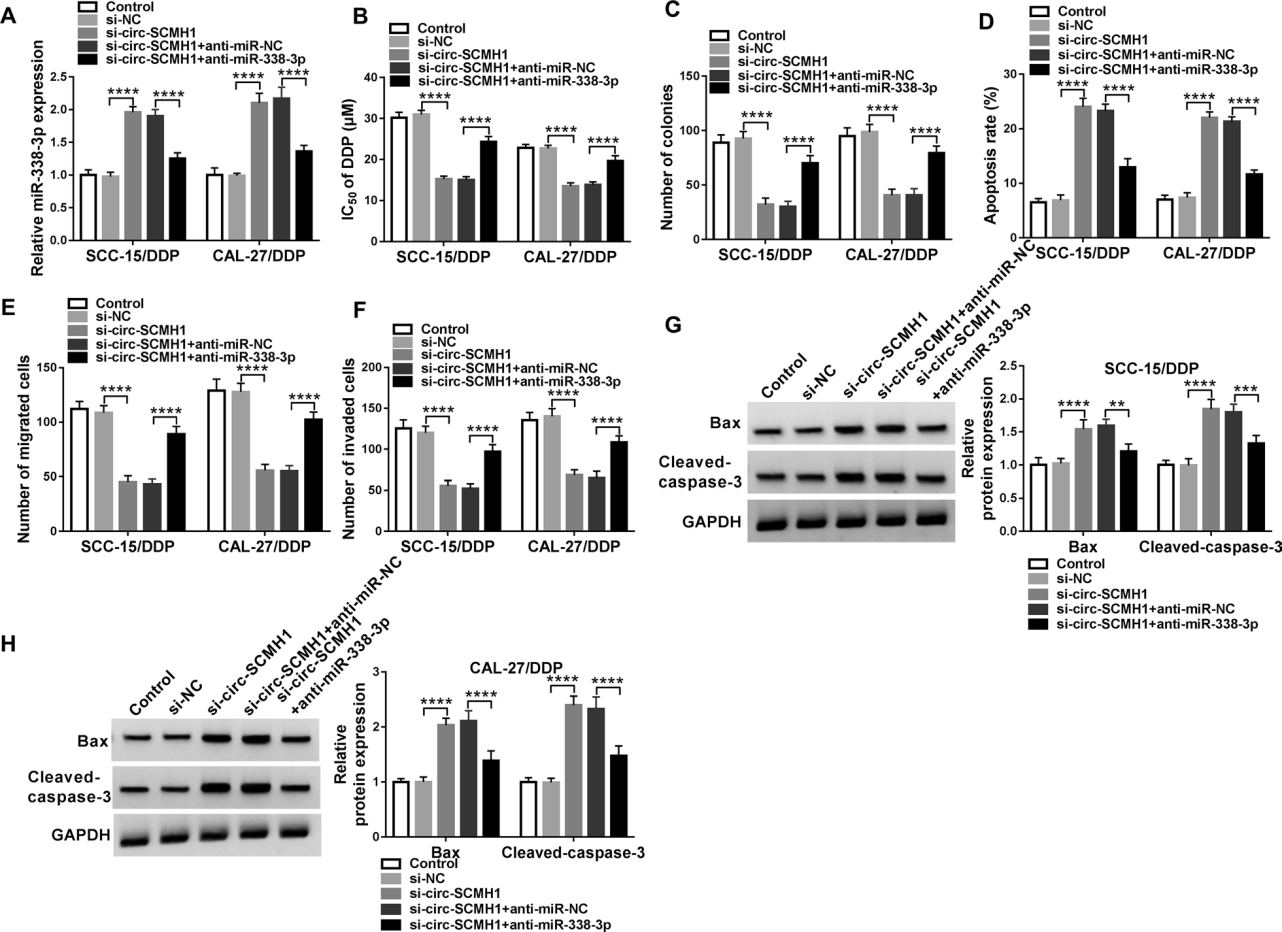
The reciprocal effect between circ-SCMH1 and miR-338-3p in DDP resistance of OSCC cells in vitro. SCC-15/DDP and CAL-27/DDP cells were transfected with si-NC alone, si-circ-SCMH1 alone or together with anti-RNA against miR-338-3p (anti-miR-338-3p) or the negative control (anti-miR-NC). **A** RT-qPCR detected miR-338-3p level after transfection with normalization to U6. **B** MTT assay identified IC_50_ of DDP after transfection. **C** Colony formation assay measured number of colonies after transfection. **D** FCM analyzed apoptosis rate after transfection. **E**, **F** Transwell assays measured numbers of migrated cells and invaded cells after transfection. **G**, **H** Western blotting examined relative protein expression of Bax and cleaved-caspase-3 with correction with GAPDH. **P < 0.01, ***P < 0.001 and ****P < 0.0001 from three separate assays

Rescue experiments were performed in SCC-15/DDP and CAL-27/DDP cells transfected with si-circ-SCMH1 alone or together with anti-miR-338-3p. RT-qPCR data showed that the upregulation of miR-338-3p mediated by circ-SCMH1 knockdown was suppressed by anti-miR-338-3p transfection (Fig. 4A). In addition, circ-SCMH1 deletion-mediated inhibition on IC_50_ of DDP and colony formation was both attenuated by miR-338-3p downregulation in SCC-15/DDP and CAL-27/DDP cells (Fig. 4B and C). Apoptosis was remarkably high in circ-SCMH1-silenced SCC-15/DDP and CAL-27/DDP cells, and this promotion was depressed by introducing anti-miR-338-3p (Fig. 4D, G, and H). The transwell migration and invasion of SCC-15/DDP and CAL-27/DDP cells were inhibited by si-circ-SCMH1 transfection, which was rescued by the presence of anti-miR-338-3p (Fig. 4E and F). These results collectively demonstrated that circ-SCMH1 deficiency might upregulate miR-338-3p to exert suppressive effect on chemoresistance of DDP-resistant OSCC cells in vitro, and blocking miR-338-3p could counteracted this effect.

### LIN28B was a downstream target of miR-338-3p

**Fig. 5:**
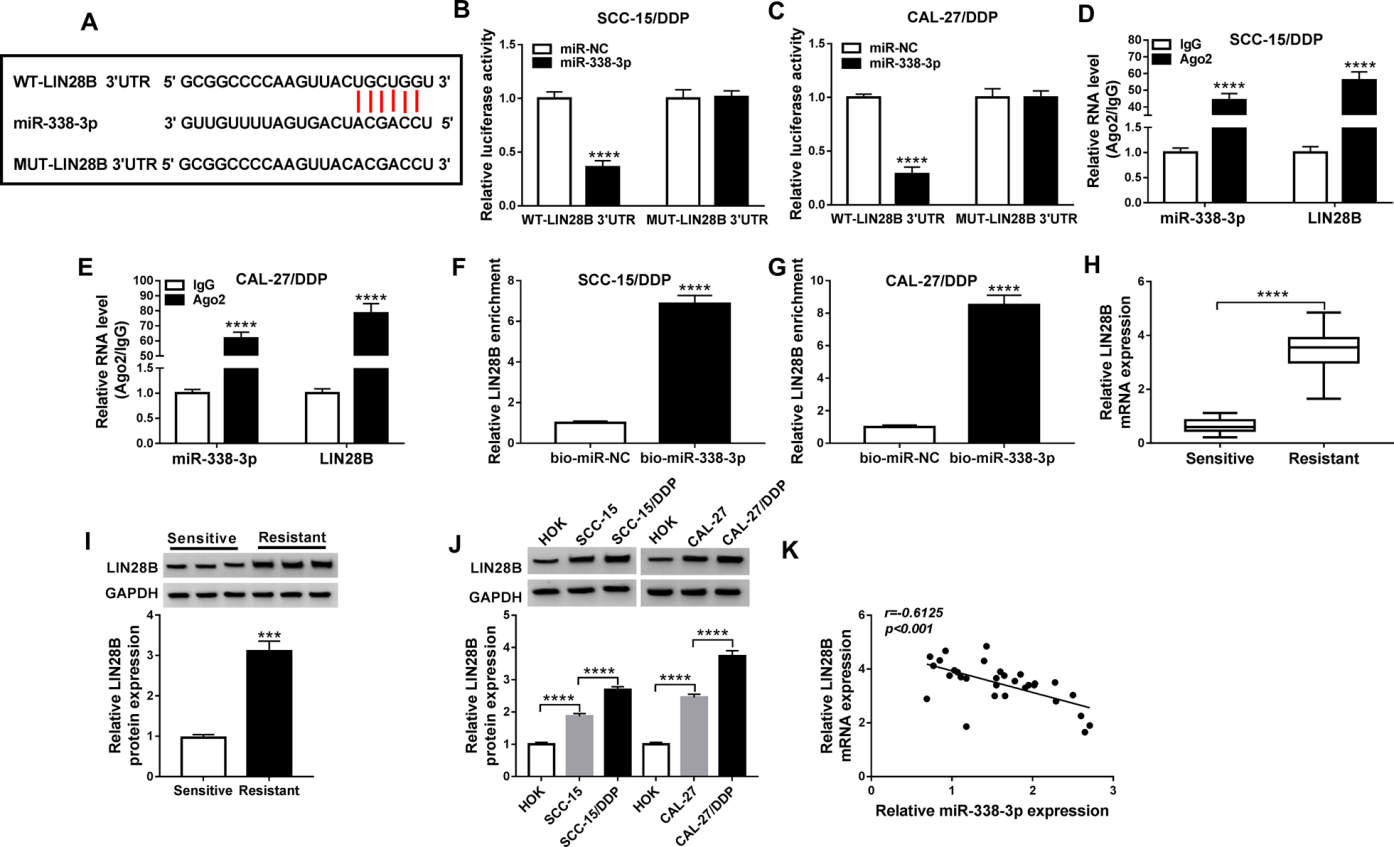
The downstream target of miR-338-3p in OSCC cells. **A** The starBase v2.0 algorithm showed the complementary binding sites among wild-type of LIN28B 3′UTR (WT-LIN28B 3′UTR), miR-338-3p and mutant-type of LIN28B (MUT-LIN28B 3′UTR). **B**, **C** Dual-luciferase reporter assay examined luciferase activity of SCC-15/DDP and CAL-27/DDP cells co-transfected with WT/MUT-LIN28B 3′UTR and miR-338-3p or miR-NC. **D**, **E** RIP assays of IgG and Ago2 examined RNA levels of miR-338-3p and LIN28B in cell extracts of SCC-15/DDP and CAL-27/DDP cells. **F**, **G** RNA pull-down assay measured LIN28B enrichment in cell extracts of SCC-15/DDP and CAL-27/DDP cells transfected with bio-miR-338-3p or bio-miR-NC. **H** RT-qPCR measured LIN28B mRNA protein expression and **I**, **J** western blotting measured LIN28B protein expression in OSCC tissues in Resistant and Sensitive groups (*N* = 31) and cell lines (HOK, SCC-15, SCC-15/DDP, CAL-27, and CAL-27/DDP) with normalization to GAPDH. **K** Pearson’s correlation (*r*) analysis evaluated the relationship between miR-338-3p and LIN28B expression in OSCC tissues in Resistant group (*N* = 31). ***P < 0.001 and ****P < 0.0001 from three separate assays

The downstream target gene of miR-338-3p was further searched and validated. According to starBase v2.0 algorithm (http://starbase/circRNA&flag/lin28b-hsa-miR-338-3p), there was a potential complementary binding sites of miR-338-3p on LIN28B 3′UTR (Fig. 5A). Luciferase activity of WT-LIN28B 3′UTR vector was consistently diminished in SCC-15/DDP and CAL-27/DDP cells co-transfected with miR-338-3p mimic (Fig. 5B and C). Furthermore, RIP assay demonstrated that miR-338-3p and LIN28B were concurrently enriched in Ago2-mediated precipitation (Fig. 5D and E), and RNA pull-down assay revealed an enrichment of LIN28B in bio-miR-338-3p-mediated precipitation (Fig. 5F and G). Expression of LIN28B at mRNA level and protein level was increased in DDP-resistant OSCC tissues (Fig. 5H and I). Additionally, LIN28B protein expression was gradually but significantly upregulated from normal HOK cells to OSCC cells (SCC-15 and CAL-27), and till to DDP-resistant cells (SCC-15/DDP and CAL-27/DDP) (Fig. 5J). There was an inverse correlation between miR-338-3p and LIN28B expression in DDP-resistant OSCC tumors (Fig. 5K). These results displayed a direct interaction between miR-338-3p and LIN28B in OSCC cells.

### Overexpression of miR-338-3p inhibited chemoresistance of DDP-resistant OSCC cells in vitro by downregulating LIN28B

**Fig. 6:**
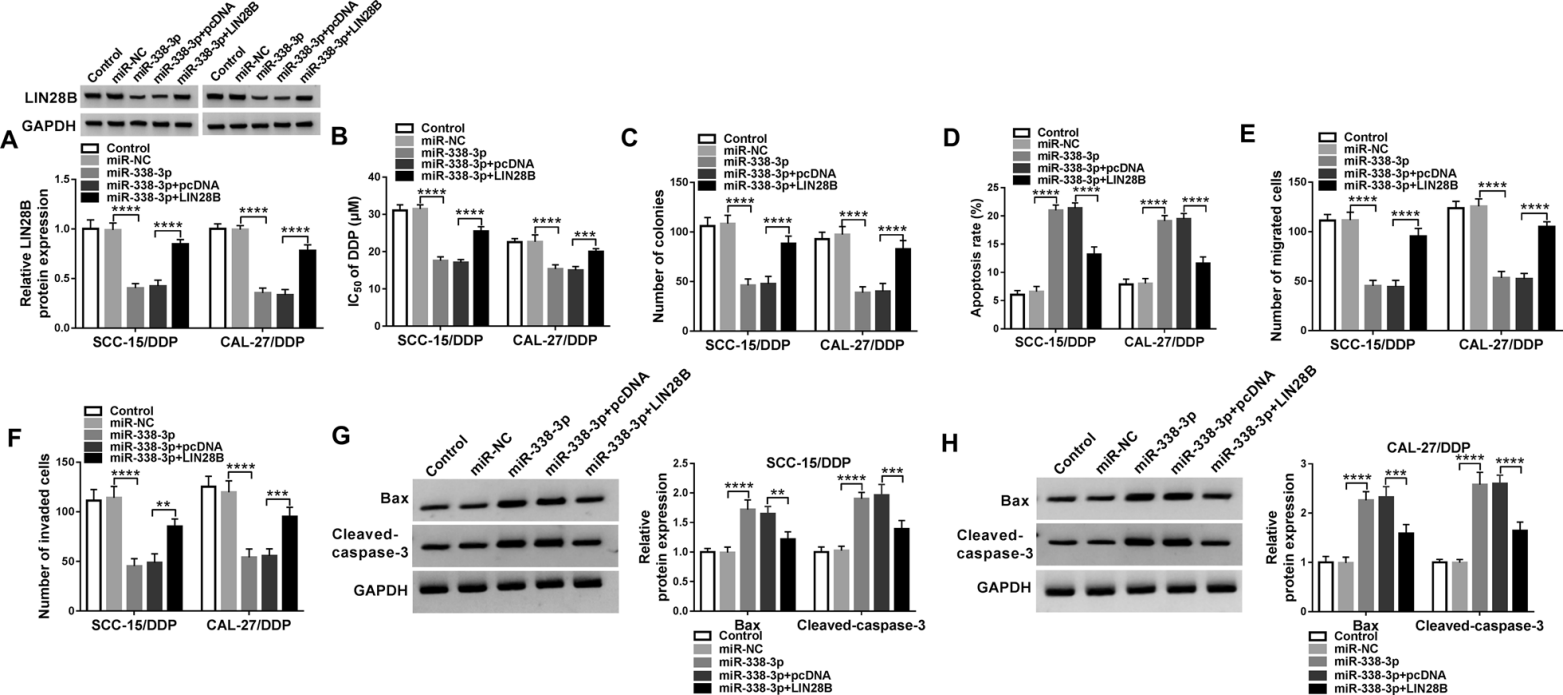
The interactive role of miR-338-3p and LIN28B in DDP resistance of OSCC cells in vitro. SCC-15/DDP and CAL-27/DDP cells were transfected with miR-NC alone, miR-338-3p alone or together with LIN28B-overexpression vector (LIN28B) or pcDNA vector. **A** Western blotting detected LIN28B protein level after transfection with normalization to GAPDH. **B** MTT assay identified IC_50_ of DDP after transfection. **C** Colony formation assay measured number of colonies after transfection. **D** FCM analyzed apoptosis rate after transfection. **E**, **F** Transwell assays measured numbers of migrated cells and invaded cells after transfection. **G**, **H** Western blotting examined relative protein expression of Bax and cleaved-caspase-3 with correction with GAPDH. **P < 0.01, ***P < 0.001 and ****P < 0.0001 from three separate assays

**Fig. 7:**
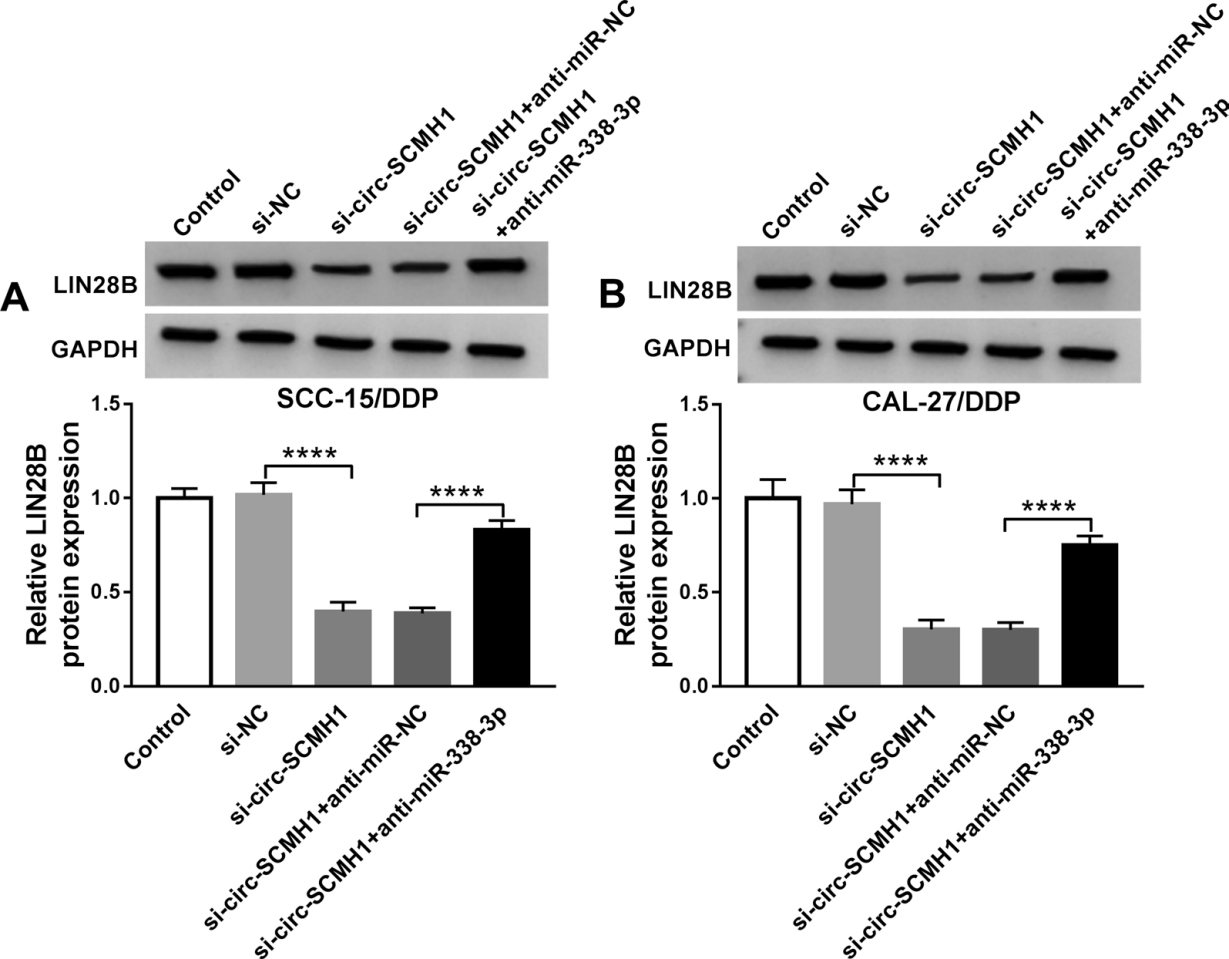
The regulation of circ-SCMH1 and miR-338-3p on LIN28B in OSCC cells. **A**, **B** Western blotting evaluated LIN28B protein level with normalization to GAPDH in SCC-15/DDP and CAL-27/DDP cells transfected with si-NC alone, si-circ-SCMH1 alone or together with anti-miR-338-3p or anti-miR-NC. ****P < 0.0001 from three separate assays

The role of both miR-338-3p and LIN28B in DDP resistance of OSCC cells in vitro was following explored. SCC-15/DDP and CAL-27/DDP cells were transfected with miR-338-3p mimic alone or together with LIN28B-overexpression vector, and a series of functional assays were carried out. The miR-338-3p mimic-transfected SCC-15/DDP and CAL-27/DDP cells exhibited low protein level of LIN28B, and LIN28B-overexpression vector rescued its expression (Fig. 6A). IC_50_ of DDP and colony formation numbers of SCC-15/DDP and CAL-27/DDP cells were suppressed after miR-338-3p mimic transfection (Fig. 6B and C). FCM data illuminated that apoptosis rate was elevated by miR-338-3p mimic-mediated downregulation of LIN28B (Fig. 6D), paralleled with increased expression of Bax and cleaved-caspase-3 (Fig. 6G and H). Transwell assays showed that numbers of migrated and invaded cells were reduced due to the introduction of miR-338-3p mimic (Fig. 6E and F). Above-mentioned data depicted a suppressive role of miR-338-3p overexpression in acquired chemoresistance in DDP-resistant OSCC cells in vitro. Notably, LIN28B restoration via pcDNA transfection partially reversed the effects of miR-338-3p overexpression in SCC-15/DDP and CAL-27/DDP cells (Fig. 6B–F). This suggested that miR-338-3p overexpression suppressed DDP resistance relying on miR-338-3p/LIN28B axis. Furthermore, western blotting data revealed that circ-SCMH1 deficiency caused inhibition on LIN28B expression in SCC-15/DDP and CAL-27/DDP cells, which was then rescued with the presence of anti-miR-338-3p (Fig. 7A and B). Therefore, a possible circ-SCMH1/miR-338-3p/LIN28B pathway was established in DDP resistance of OSCC cells.

### Knockdown of circ-SCMH1 restrained tumor growth and DDP resistance of OSCC cells in vivo

**Fig. 8:**
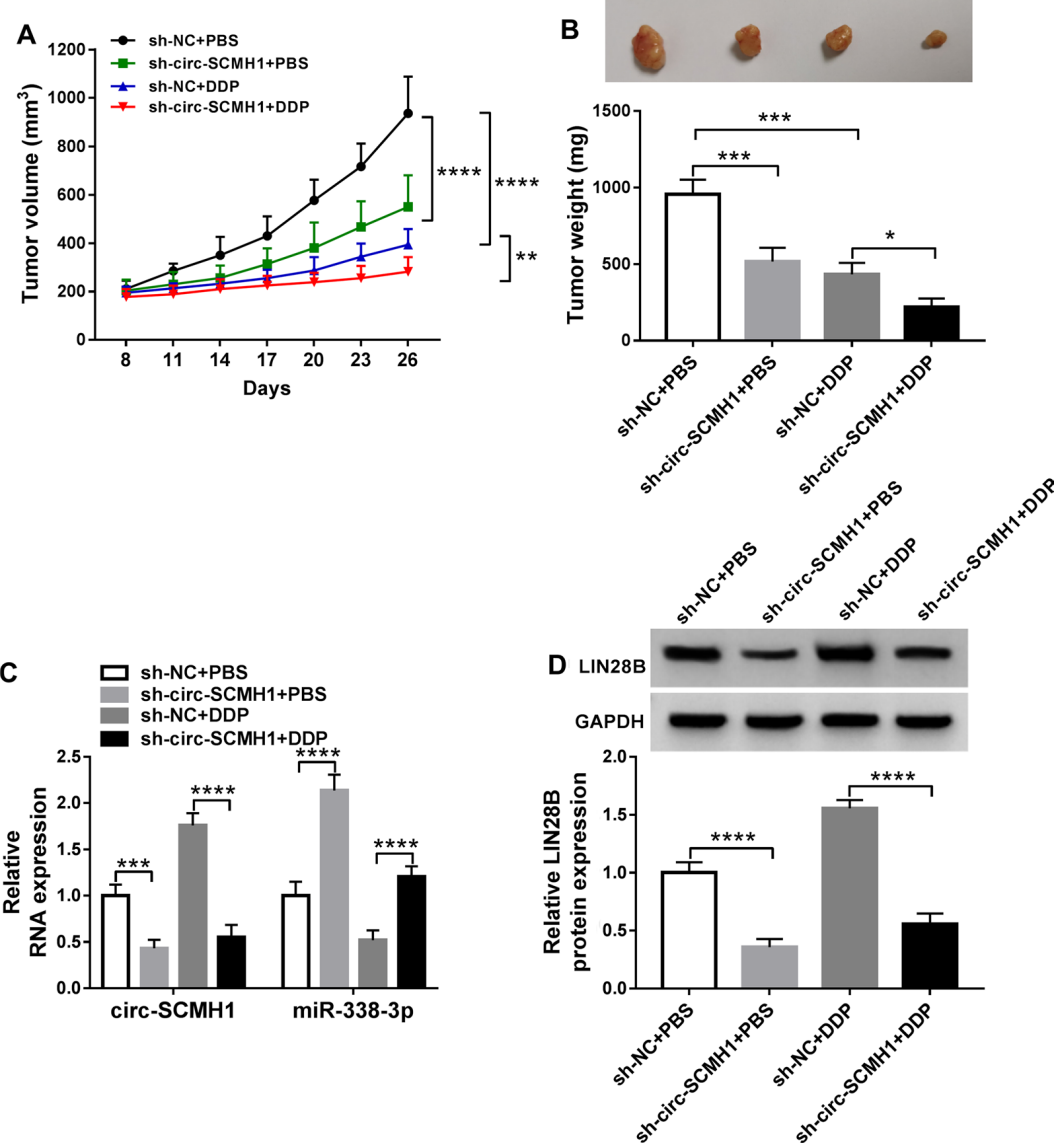
Role of circ-SCMH1 in DDP resistance of OSCC cells in vivo. Xenograft tumor models were established using SCC-15/DDP cells stably transfected with shRNA against circ-SCMH1 (sh-circ-SCMH1) or the negative control or (sh-NC), and then treated with DDP (3 mg/kg) or equal volume of phosphate-buffered saline (PBS). **A**, **B** Tumor volume and weight were measured using caliper and electronic balance. **C** RT-qPCR measured circ-SCMH1 and miR-338-3p levels in tumor tissues of xenograft mice with normalization to GAPDH and U6, respectively. **D** Western blotting measured LIN28B and GAPDH protein level in tumor tissues of xenograft mice. *P < 0.05, **P < 0.01, ***P < 0.001, and ****P < 0.0001 from three separate assays

In vivo, xenograft tumor models were utilized to measure tumor growth of SCC-15/DDP cells in nude mice. As shown in Fig. 8A and B, the volume and weight of xenograft tumors were lowered by sh-circ-SCMH1 transfection no matter with or without DDP treatment. Molecularly, sh-circ-SCMH1 transfection led to a downregulation of circ-SCMH1 and LIN28B in xenograft tumors, as well as an upregulation of miR-338-3p (Fig. 8C and D). This outcome suggested a suppressive role of circ-SCMH1 deficiency in tumor growth and chemoresistance in DDP-resistant OSCC cells in vivo by modulating miR-338-3p and LIN28B.

### EVs mediated the transport of circ-SCMH1

**Fig. 9:**
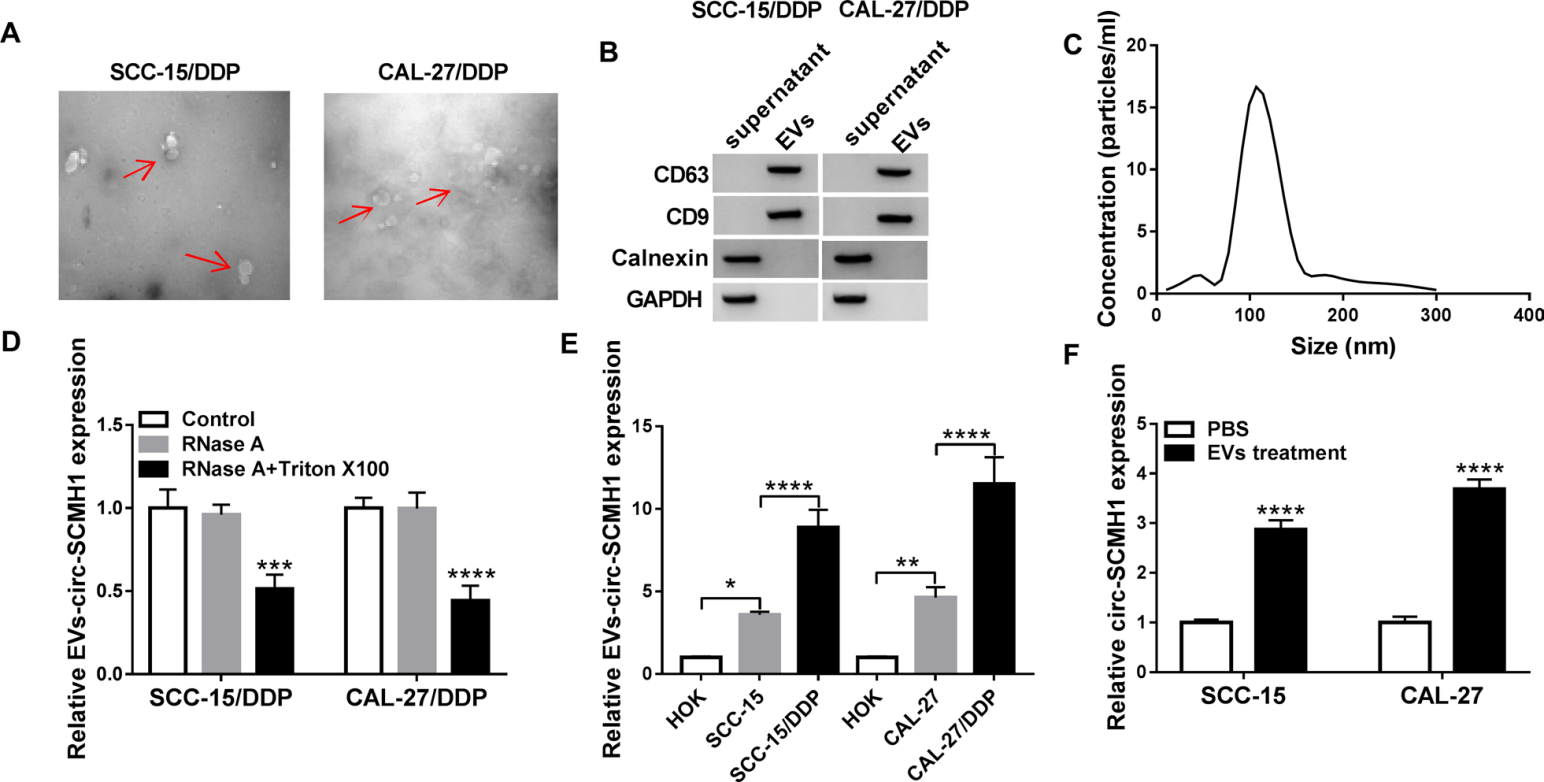
Circ-SCMH1 was carried and secreted by EVs. EVs were isolated from SCC-15/DDP and CAL-27/DDP cells. **A** Transmission electron microscopic (TEM) analysis examined the morphology and size of isolated EVs. **B** Western blotting measured protein expression of CD63, CD9, Calnexin and GAPDH. **C** Nanoparticle Tracking Analysis (NTA) software tracked the concentration and size of isolated EVs. **D–F** RT-qPCR detected **D** circ-SCMH1 level in EVs ( EVs-circ-SCMH1) in SCC-15/DDP and CAL-27/DDP cells-derived EVs treated with RNase A alone or combined with Triton X100, and **E** EVs-circ-SCMH1 level in HOK, SCC-15, SCC-15/DDP, CAL-27, and CAL-27/DDP cells, and **F** circ-SCMH1 level in SCC-15 and CAL-27 cells treated with SCC-15/DDP and CAL-27/DDP cells-derived EVs or PBS. *P < 0.05, **P < 0.01 and ****P < 0.0001 from three separate assays

CircRNAs in human saliva, blood and exosomes could be cancer biomarkers and potential therapeutics of OSCC [27, 28]. Thus, we wondered whether circ-SCMH1 was one cargo of exosomes isolated from DDP-resistant OSCC cells. TEM showed a spherical structure of EVs from both SCC-15/DDP and CAL-27/DDP cells (Fig. 9A). Western blotting identified high expression of CD63 and CD9 (surface markers of exosomes [29]) in these EVs without cell debris contamination (Calnexin expression) (Fig. 9B). NTA software showed that these EVs were 110–150 nm particles (Fig. 9C). Moreover, expression of circ-SCMH1 in EVs (EVs-circ-SCMH1) was not altered by RNase A treatment, but dramatically impaired with RNase A and Triton X100 co-treatment (Fig. 9D). EVs-circ-SCMH1 expression was stepwise increased from normal HOK cells to DDP-sensitive OSCC cells (SCC-15 and CAL-27), and till to DDP-resistant OSCC cells (Fig. 9E). These data suggested that circ-SCMH1 was an EVs cargo, and EVs-circ-SCMH1 was associated with acquired DDP resistance in OSCC cells. Furthermore, EVs treatment could induce parental cells to express higher cellular circ-SCMH1 (Fig. 9F). These results indicated that circ-SCMH1 could be carried and transferred via EVs in DDP-resistant OSCC cells.

## Discussion

Recently, several circRNAs had been declared to be oncogenic factors in OSCC, such as circ_0000199 [30], circ_0109291 [31], circ_0001821 [32], circ_0013339 [33], and circ_0011946 [18]. Here, we validated these circRNAs expression in this cohort of OSCC tissues, and there were three circRNAs including circ_0109291, circ_0001821 and circ-SCMH1 that were upregulated in DDP-resistant OSCC tissues. Here, we further investigated the role of circ-SCMH1 (the most upregulated one) in DDP resistance and malignant progression in OSCC.

We observed an upregulation of circ-SCMH1 in DDP-resistant OSCC tissues and cells than DDP-sensitive OSCC tissues and cells. These suggested that circ-SCMH1 expression might be associated with OSCC tumorigenesis and DDP resistance. Functionally, knockdown of circ-SCMH1 via transfection induced inhibition of colony formation and migration/invasion of SCC-15/DDP and CAL-27/DDP cells in vitro, which was similarly illustrated by Meng et al*.* [18] in CAL-27 cells. Besides, cell viability and wound-healing ability of CAL-27 cells were suppressed by silencing circ-SCMH1 [18]. The suppressive effect of circ-SCMH1 on cell proliferation, epithelial–mesenchymal transition (EMT), and migration/invasion was also found in breast cancer and hepatocellular carcinoma cells [16, 17]. This present study revealed an inhibition of circ-SCMH1 deficiency on IC_50_ of DDP and cell growth in DDP-resistant OSCC cells, accompanied with apoptosis promotion. Above-mentioned evidences could propose circ-SCMH1 as a novel oncogene in human cancers and DDP resistance, and this hypothesis remained to be further validated in many different types of malignant tumors.

Notably, we also noticed that circ-SCMH1 was upregulated in EVs from SCC-15/DDP and CAL-27/DDP cells; EVs treatment could increase cellular expression of circ-SCMH1 in SCC-15 and CAL-27. In consideration of the presence of exosomes in saliva as well [28], circ-SCMH1 in salivary exosomes could show greater application prospect. These observations might imply a potentiality of exosomal circ-SCMH1 from saliva as noninvasive biomarker to distinguish healthy controls, DDP-sensitive OSCC and DDP-resistant OSCC. By the way, circ-SCMH1 high expression might predict a high TNM stage and metastasis in OSCC patients. Mechanically, circ-SCMH1/miRNAs/mRNAs co-expression network was predicted and constructed in breast cancer cells [16]; whereas, Meng et al*.* [18] did not further identify this classic regulatory mechanism. Thus, this study might provide the first ceRNA pathway of circ-SCMH1 in OSCC, namely circ-SCMH1/miR-338-3p/LIN28B axis.

MiR-338-3p was a tumor suppressor in various human cancers [34–36] including OSCC [37]. Expression of miR-338-3p was previously declared to be inhibited in OSCC tumors and cells [37, 38], and miR-338-3p could negatively modulate proliferation, colony formation, and migration/invasion of OSCC cells both in vitro and in vivo. In this study, we considered a downregulation of miR-338-3p in DDP-resistant OSCC tissues and cells; overexpressing miR-338-3p functioned an anti-tumor role in DDP-resistant OSCC cells in vitro by inhibiting DDP resistance. In addition, this promoting role of miR-338-3p in DDP sensitivity was analogous to that in ovarian cancer and nasopharyngeal cancer [39, 40]. This study might be a pioneer to discuss the suppression of miR-338-3p on DDP resistance in OSCC.

LIN28B was verified as a novel downstream target gene of miR-338-3p, and thus participated in DDP resistance of OSCC. Zhang et al. [41] manifested that expression of LIN28B was upregulated in OSCC tumors and cell lines, which was also demonstrated in studies of Wang et al. [22] and Lin et al. [42]. Here, we further discovered the upregulation of LIN28B in DDP-resistant OSCC tissues and cells, and LIN28B high level could counteract DDP resistance of SCC-15/DDP and CAL-27/DDP cells with miR-338-3p overexpression. Similarly, LIN28B mediated the inhibitory effect on cell proliferation, migration and EMT of SCC-15 cells [41]. By the way, an upregulation of LIN28B was also observed in metastatic lymph node [42]; deleting LIN28B could attenuate oncogenicity of human gingival epithelial cell line (SG) and hypopharyngeal squamous cell carcinoma cell line (FaDu) [42], and both of which were OSCC cells. Moreover, cancer stem cells (CSCs) were one essential reason of cancer progression and chemoresistance, and LIN28B was shown to be the highest gene in CSC-like cells [43]. Ectopic expression of LIN28B induced OSCC cells exhibiting CSC-like properties by regulating let-7, Oct4 and Sox2 [43, 44]. Therewith, LIN28B was identified to be associated with OSCC tumor size, advanced clinical stage, shorter disease-free survival, and lower overall survival in clinic [22, 45]. More importantly, LIN28B had two isoforms: LIN28B-long and -short isoforms [46]; LIN28B-long was related to drug resistance to 5-fluorouracil and DDP through upregulating ERCC1, and LIN28B-short seemed to play antagonistic functions to the long isoform [47]. However, the difference between the both isoforms was not further investigated in DDP resistance of OSCC in this study.

In conclusion, we demonstrated the dysregulation and role of circ-SCMH1, miR-338-3p and LIN28B in DDP-resistant OSCC tissues and cells. Circ-SCMH1, as an oncogene, might contribute to the malignant development and DDP resistance of OSCC cells both in vitro and in vivo via EVs secretion and circ-SCMH1/miR-338-3p/LIN28B pathway.

## Supplementary Information

**Additional file 1:** **Figure S1.** Dysregulation of circRNAs in OSCC patients. RT-qPCR detected relative RNA expression of hsa_circ_0000199 (circ_0000199), circ_0109291, circ_0001821, circ_0013339, and circ_0011946 in OSCC tissues in resistant and sensitive groups (N=31). *P<0.05, **P<0.01, ***P<0.001, and ****P<0.0001 from three separate assays.

**Additional file 2:** **Figure S2.** Expression of circ-SCMH1 in DDP-resistant OSCC tissues and cells. RT-qPCR detected circ-SCMH1 level with normalization to β-actin in (A) Resistant (N=31) and Sensitive (N=31) tissues, and (B) SCC-15/DDP, CAL-27/DDP, SCC-15, CAL-27, and HOK cells, (C, D) circ-SCMH1 and linear SCMH1 expression in SCC-15/DDP and CAL-27/DDP cells after RNase R treatment or Mock treatment, and (E, F) circ-SCMH1, U6 and GAPDH expression in the cytoplasm and nucleus of SCC-15/DDP and CAL-27/DDP cells. **P<0.01, ***P<0.001, and ****P<0.0001 from three separate assays.

**Additional file 3:** **Figure S3.** Silencing circ-SCMH1 could not alter host gene expression. RT-qPCR detected relative SCMH1 mRNA expression in SCC-15/DDP and CAL-27/DDP cells transfected with si-NC or si-circ-SCMH1, compared to that in control cells (without transfection).

**Additional file 4:** **Figure S4.** The expression model of circ-SCMH1 and miR-338-3p in SCC-15/DDP and CAL-27/DDP cells after transfection. SCC-15/DDP and CAL-27/DDP cells were transfected with si-circ-SCMH1 or miR-338-3p mimic. (A and B) The expression of circ-SCMH1 (A) and miR-338-3p (B) was detected by RT-qPCR.

**Additional file 5: Figure S5.** Silencing of circ-SCMH1 suppressed cell progression of DDP-sensitive OSCC cells in vitro. SCC-15 and CAL-27 cells were transfected with si-circ-SCMH1 or si-NC, comparing to control cells (without transfection). (A) PCR-qRT detected relative circ-SCMH1 expression after transfection with normalization to GAPDH. (B) MTT assay identified cell viability after transfection. (C) Colony formation assay measured number of colonies after transfection. (D) FCM analyzed apoptosis rate after transfection. (E, F) Transwell assays measured numbers of migrated cells and invaded cells after transfection. **P<0.01, ***P<0.001 and ****P<0.0001 from three separate assays.

## Data Availability

The datasets generated during and/or analysed during the current study are available from the corresponding author on reasonable request.
