# Correction to: Blocking circ‑SCMH1 (hsa_circ_0011946) suppresses acquired DDP resistance of oral squamous cell carcinoma (OSCC) cells both in vitro and in vivo by sponging miR‑338‑3p and regulating LIN28B

**DOI:** 10.1186/s12935-022-02491-4

**Published:** 2022-02-06

**Authors:** Feng Qiu, Bin Qiao, Nan Zhang, Zheng Fang, Lu Feng, Shanfeng Zhang, Weiliu Qiu

**Affiliations:** 1 Department of Stomatology, The First Affiliated Hospital of Zhengzhou University, Zhengzhou, 450052 Henan China; 2 School of Basic Medical Sciences, Zhengzhou University, Zhengzhou, 450001 Henan China; 3 Experimental Center for Basic Medicine, Biochemistry and Molecular Biology, Zhengzhou University, Zhengzhou, 450000 Henan China; 4 Department of Oral and Maxillofacial Surgery, School of Medicine, Ninth People’s Hospital, Shanghai Jiao Tong University, No. 639, Manufacturing Bureau Road, Shanghai, 200011 China

## Correction to: Cancer Cell Int (2021) 21:412 https://doi.org/10.1186/s12935-021-02110-8

In this article [1], the affiliation “The First Affiliated Hospital of Zhengzhou University” for the last corresponding author Weiliu Qiu was missing. The affiliations are corrected with this correction.
