# Supplementary figures and images for: Blocking circ-SCMH1 (hsa_circ_0011946) suppresses acquired DDP resistance of oral squamous cell carcinoma (OSCC) cells both in vitro and in vivo by sponging miR-338-3p and regulating LIN28B
Source: Cancer Cell Int. 2021 Aug 5;21:412. doi: 10.1186/s12935-021-02110-8 (PMC8340538; doi:10.1186/s12935-021-02110-8)

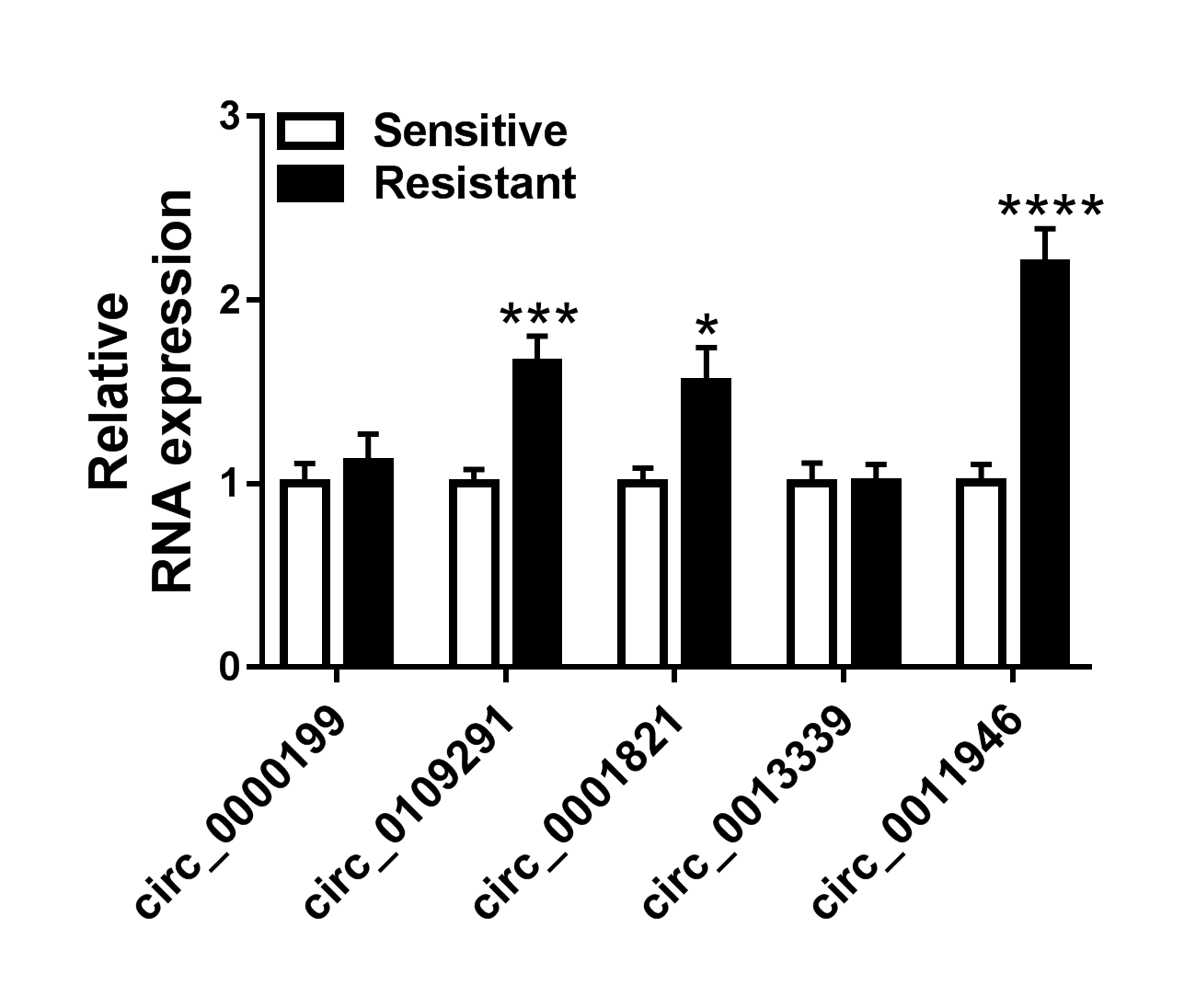

Supplement: Supplementary file 1 — Additional file 1: Figure S1. Dysregulation of circRNAs in OSCC patients. RT-qPCR detected relative RNA expression of hsa_circ_0000199 (circ_0000199), circ_0109291, circ_0001821, circ_0013339, and circ_0011946 in OSCC tissues in resistant and sensitive groups (N=31). *P<0.05, **P<0.01, ***P<0.001, and ****P<0.0001 from three separate assays. [file 12935_2021_2110_MOESM1_ESM.tif]

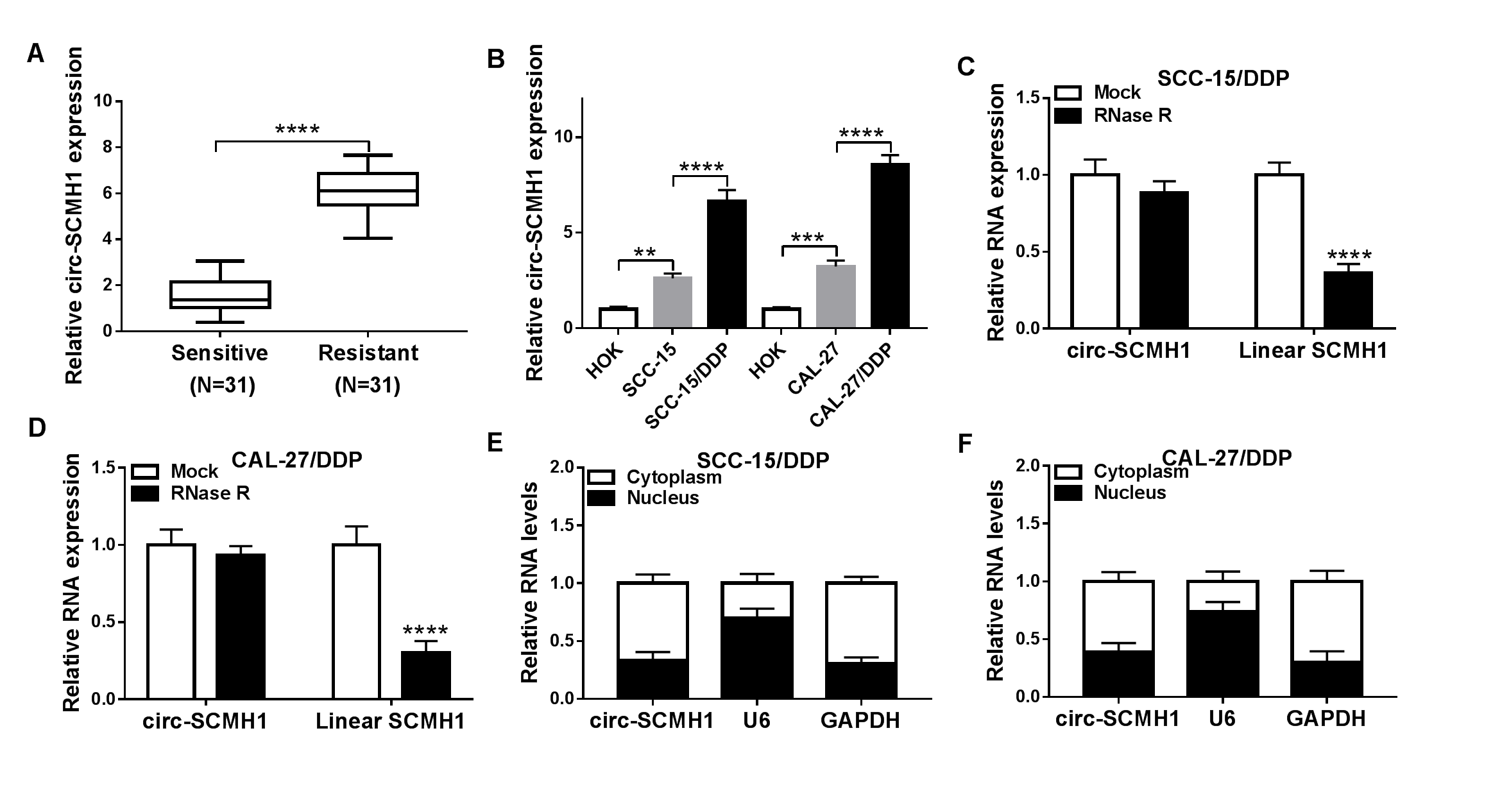

Supplement: Supplementary file 2 — Additional file 2: Figure S2. Expression of circ-SCMH1 in DDP-resistant OSCC tissues and cells. RT-qPCR detected circ-SCMH1 level with normalization to β-actin in (A) Resistant (N=31) and Sensitive (N=31) tissues, and (B) SCC-15/DDP, CAL-27/DDP, SCC-15, CAL-27, and HOK cells, (C, D) circ-SCMH1 and linear SCMH1 expression in SCC-15/DDP and CAL-27/DDP cells after RNase R treatment or Mock treatment, and (E, F) circ-SCMH1, U6 and GAPDH expression in the cytoplasm and nucleus of SCC-15/DDP and CAL-27/DDP cells. **P<0.01, ***P<0.001, and ****P<0.0001 from three separate assays. [file 12935_2021_2110_MOESM2_ESM.tif]

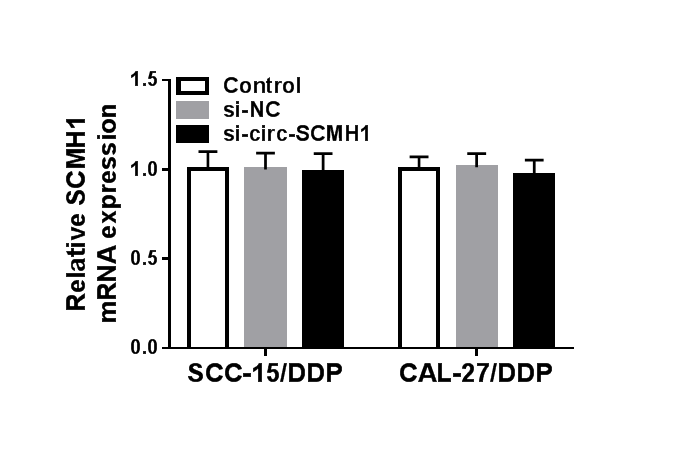

Supplement: Supplementary file 3 — Additional file 3: Figure S3. Silencing circ-SCMH1 could not alter host gene expression. RT-qPCR detected relative SCMH1 mRNA expression in SCC-15/DDP and CAL-27/DDP cells transfected with si-NC or si-circ-SCMH1, compared to that in control cells (without transfection). [file 12935_2021_2110_MOESM3_ESM.tif]

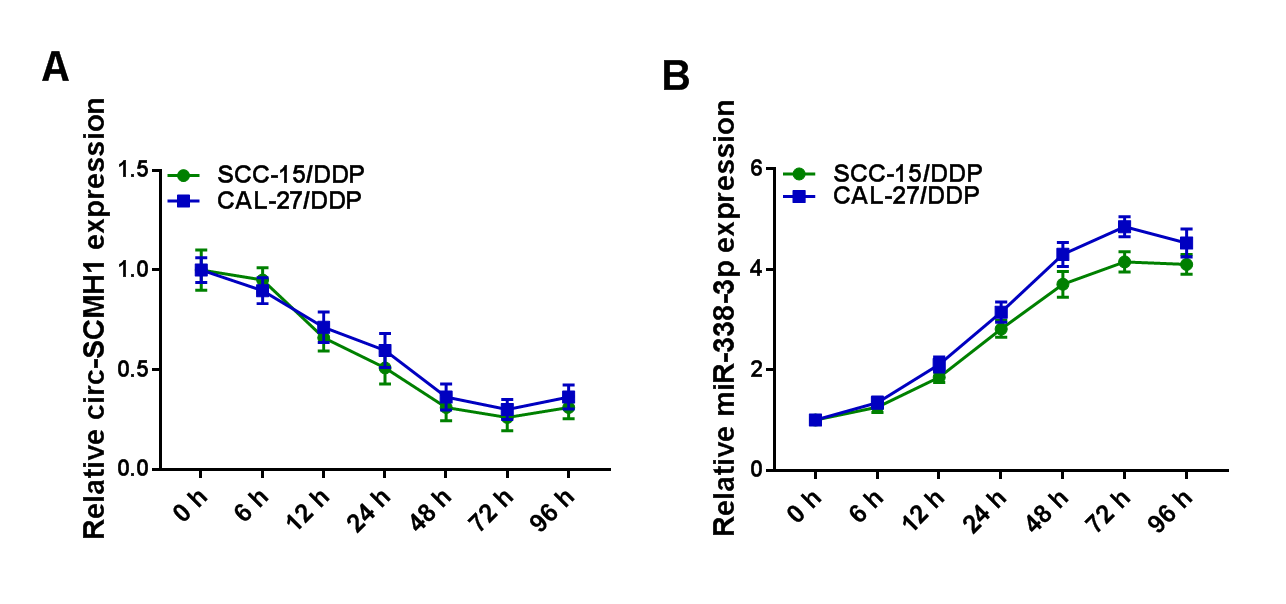

Supplement: Supplementary file 4 — Additional file 4: Figure S4. The expression model of circ-SCMH1 and miR-338-3p in SCC-15/DDP and CAL-27/DDP cells after transfection. SCC-15/DDP and CAL-27/DDP cells were transfected with si-circ-SCMH1 or miR-338-3p mimic. (A and B) The expression of circ-SCMH1 (A) and miR-338-3p (B) was detected by RT-qPCR. [file 12935_2021_2110_MOESM4_ESM.tif]

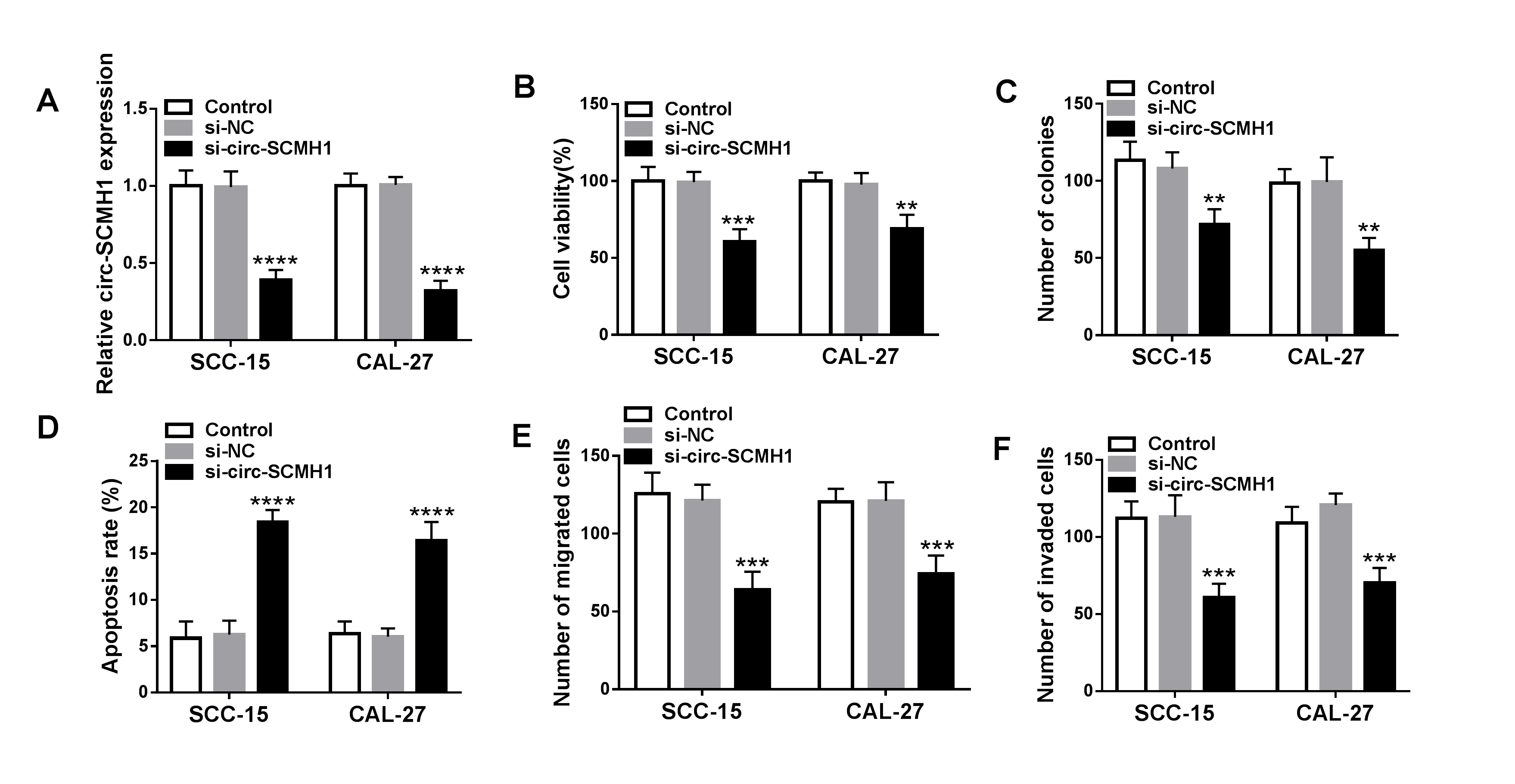

Supplement: Supplementary file 5 — Additional file 5: Figure S5. Silencing of circ-SCMH1 suppressed cell progression of DDP-sensitive OSCC cells in vitro. SCC-15 and CAL-27 cells were transfected with si-circ-SCMH1 or si-NC, comparing to control cells (without transfection). (A) PCR-qRT detected relative circ-SCMH1 expression after transfection with normalization to GAPDH. (B) MTT assay identified cell viability after transfection. (C) Colony formation assay measured number of colonies after transfection. (D) FCM analyzed apoptosis rate after transfection. (E, F) Transwell assays measured numbers of migrated cells and invaded cells after transfection. **P<0.01, ***P<0.001 and ****P<0.0001 from three separate assays. [file 12935_2021_2110_MOESM5_ESM.tif]
